# Sorafenib and locoregional deep electro-hyperthermia in advanced hepatocellular carcinoma: A phase II study

**DOI:** 10.3892/ol.2014.2376

**Published:** 2014-07-24

**Authors:** GENNARO GADALETA-CALDAROLA, STEFANIA INFUSINO, IDA GALISE, GIROLAMO RANIERI, GIANLUCA VINCIARELLI, VITO FAZIO, ROSA DIVELLA, ANTONELLA DANIELE, GIANFRANCO FILIPPELLI, COSMO DAMIANO GADALETA

**Affiliations:** 1 Interventional Radiology and Medical Oncology Unit, National Cancer Research Centre, National Cancer Institute ‘Giovanni Paolo II’, Bari 70124, Italy; 2 Medical Oncology Unit, ‘S. Francesco di Paola’ Hospital, Via Promintesta, Paola 87027, Italy; 3 Apulia Cancer Registry, Statistic and Epidemiology Unit, National Cancer Research Centre, National Cancer Institute ‘Giovanni Paolo II’, Bari 70124, Italy; 4 Clinical Pathology Laboratory, National Cancer Research Centre, National Cancer Institute ‘Giovanni Paolo II’, Bari 70124, Italy

**Keywords:** carcinoma, electro-hyperthermia, hepatocellular, sorafenib

## Abstract

The standard treatment for advanced hepatocellular carcinoma (HCC) is sorafenib, a multikinase inhibitor of tumor cell proliferation and angiogenesis. Hyperthermia inhibits angiogenesis and promotes apoptosis. Potential synergic antiangiogenic and proapoptotic effects represent the rationale for combining sorafenib with electro-hyperthermia (EHY) in HCC. A total of 21 patients (median age, 64 years; range, 55–73 years) with advanced HCC were enrolled in the current study between February 2009 and September 2010. EHY was achieved by arranging capacitive electrodes with a deep hypothermia radiofrequency field of 13.56 Mhz at 80 W for 60 min, three times per week for six weeks, followed by two weeks without treatment, in combination with sorafenib at a dose of 800 mg every other day. According to the modified Response Evaluation Criteria in Solid Tumors criteria, 50% achieved stable disease, 5% achieved partial response and 45% achieved progressive disease. No complete response was observed. The progression-free survival (PFS) rate at six months was 38%, while the median PFS and overall survival times were 5.2 [95% confidence interval (CI), 4.2–6.2) and 10.4 (95% CI, 10–11) months, respectively. The overall incidence of treatment-related adverse events was 80%, predominantly of grade 1 or 2. Grade 3 toxicity included fatigue, diarrhea, hand-foot skin reaction and hypertension. In the present study, the sorafenib plus EHY combination was feasible and well tolerated, and no major complications were observed. The initial findings indicated that this combination offers a promising option for advanced HCC.

## Introduction

Hepatocellular carcinoma (HCC) is the sixth most common type of neoplasm and the third most frequent cause of cancer-related mortality in Western countries (1). The Barcelona Clinic Liver Cancer (BCLC) strategy is a classification which stratifies patients according to prognosis, providing a link to treatment. Patients with advanced HCC (BCLC stage C) exhibit cancer-related symptoms [symptomatic tumors; Eastern Cooperative Oncology Group (ECOG) grades 1–2], including macrovascular invasion (segmental or portal invasion) or extrahepatic spread (lymph node involvement or metastases) which carry a poor prognosis, with a predicted median survival time of six months or survival rate of 25% at one year. The standard treatment for advanced HCC (BCLC stage C) is sorafenib, an oral multikinase inhibitor that inhibits the following: i) Serine-threonine kinases, Raf-1 and B-Raf, of the Raf/MEK/ERK signaling pathway; and ii) the receptor tyrosine kinase activity of VEGFR1,2 and 3, PDGFR-β, c-Kit, Flt-3 and RET (2,3). Sorafenib inhibits tumor cell proliferation and tumor angiogenesis, and increases the rate of apoptosis in a number of tumors (4). In two randomized clinical trials [Sorafenib HCC Assessment Randomized Protocol Trial (SHARP) and Asia Pacific Liver Cancer Study], sorafenib treatment resulted in longer median survival time and time to progression (TTP) in advanced hepatocellular cancer when compared with the placebo (5,6).

Electro-hyperthermia (EHY), also known as oncothermia or extracellular hyperthermia, is a method of locoregional hyperthermia, established by the direct absorption of an electric field energy in the extracellular liquid with a subsequent temperature gradient between the extra- and intracellular compartments; this gradient destroys cancer cell membranes, leading to necrosis or apoptosis. As the conductivity and the dielectric constant of the extracellular matrix in malignant tissue are higher than in the normal tissue, this technique results in selective tumor tissue destruction. For this reason, energy absorption at the applied frequency is significantly increased. Furthermore, malignant cells typically exhibit relatively rigid membranes due to increased phospholipid concentrations, therefore, EHY is likely to selectively destroy malignant cells prior to affecting the healthy cells. EHY increases apoptosis (producing membrane heat shock proteins), blocks further proliferation, terminates tumor cell dissemination (re-establishing the adherent connections) and increases immunogenicity. EHY is a complementary treatment in various types of tumors, such as brain, soft tissue, liver and abdominal, pancreatic, and head and neck tumors (7).

Hyperthermia inhibits angiogenesis through endothelial cell (EC) damage and increases PAI-1 genetic expression in ECs (8). Several pharmacodynamics (including the acceleration of the primary mode of action and an increased intracellular drug concentration) and pharmacokinetics (for example drug uptake, distribution, metabolism and excretion) interactions have been described between drugs and temperature. The cytotoxic effect of the majority of alkylating agents (including cyclophosphamide and ifosfamide) and platinum compounds are linearly enhanced with increasing temperature from 37 to >40°C. Conversely, doxorubicin appears to have a defined temperature threshold, whilst the majority of antimetabolites (such as 5-fluorouracil), as well as vinca alkaloids and taxanes, show no dependency to hyperthermia (9). In certain animal models, several drugs (including KB-R8498, flavone acetic acid, vinblastine and combretastatin) have been observed to induce a temporary reduction in tumor blood, but only in combination with hyperthermia significant tumor responses (10).

The potential synergic antiangiogenic and proapoptotic effects are the rationale for combining sorafenib and EHY for the treatment of HCC (11,12). The present study evaluated the efficacy and safety of this combination in patients with advanced HCC in a phase II study at the National Cancer Institute ‘Giovanni Paolo II’ (Bari, Italy).

## Materials and methods

A mono-institutional uncontrolled phase II trial was conducted on advanced HCC patients. The Ethical Committee of the National Cancer Institute ‘Giovanni Paolo II’ approved the protocol which was in accordance with the ethical guidelines of the 1975 Declaration of Helsinki. Written informed consent was obtained from each patient.

### Patient eligibility

Between February 2009 and September 2010, 21 patients, comprising 14 (67%) males and seven (33%) females with a median age of 64 years (range, 55–73 years), were enrolled in this study at the at the National Cancer Institute ‘Giovanni Paolo II’. Patients with measurable, histologically confirmed and inoperable HCC who had not received prior systemic treatment for HCC were eligible for enrollment. The inclusion criteria included age of ≥18 years; Eastern Cooperative Oncology Group performance status of ≤2; Child-Pugh (CP) score of A or B; life expectancy of ≥12 weeks; adequate hematological status (platelet count of ≥60×10^9^/l; hemoglobin level of ≥ 8.5 g/dl; and prothrombin time international normalized ratio of ≤2.3 or prothrombin time of ≤6 sec above the control); adequate liver function tests (albumin level of ≥2.8 g/dl, total bilirubin level of ≤3 mg/dl and alanine aminotransferase and aspartate aminotransferase levels of ≤5 times the upper limit of the normal range) and adequate renal function tests (serum creatinine level of ≤1.5 times the upper limit of the normal range). Hepatitis B virus (HBV) or hepatitis C virus (HCV) infection status at baseline were collected from the medical history or laboratory tests. The patients were required to have at least one untreated target lesion that could be measured in one dimension, according to the Response Evaluation Criteria in Solid Tumors (RECIST) (13).

### Treatment and dose modifications

Patients received 400 mg sorafenib twice a day and EHY with capacitative electrodes with a deep hypothermia radiofrequency field of 13.56 Mhz at 80 W for 60 min, three times a week for six weeks, followed by two weeks without treatment. Regional hyperthermia and thermal mapping were performed according to the European Society of Hyperthermic Oncology guidelines for quality and safety assurance (14). Locoregional deep-hyperthermia was performed using the Oncotherm EHY-2000 medical device (Oncotherm GmbH, Traisdorf, Germany). A large, water-cooled bolus asymmetric electrode (30 cm in diameter) was used.

Sorafenib treatment interruptions and dose reductions (initially 200 mg twice daily, then reduced to 200 mg once daily) were allowed for drug-related toxicity, measured according to the National Cancer Institute Common Toxicity Criteria (v 3.0) (15).

Patients with dermatologic toxicities of grade 3/4 and patients with hematological toxicity of grade 3 received lower doses. A dose delay was introduced for grade 4 hematologic toxicities and grade 3 non-hematologic toxicities, until toxicity was grade 2 or less; patients were then treated at one dose level lower and therapy was discontinued if recovery time was three weeks or longer. Patients with drug-related grade 4 non-hematologic toxicities were removed from the study.

For hand-foot skin reaction (HFSR), dose modifications based on prescribing information and 2008 consensus panel recommendations were used (16).

Treatment was continued until disease progression (PD) or unacceptable drug-related toxicities.

### Response assessment

Bidimensional tumor measurements were performed at baseline and every eight weeks (one cycle), according to RECIST, by computed tomography or magnetic resonance imaging. Throughout the study, the lesions were measured at baseline and evaluated using the same technique. Overall tumor response was scored as a complete response (CR), partial response (PR) or stable disease (SD) if the response was confirmed at least four weeks later. The disease control rate (DCR) was the proportion of patients who had the best response rating of CR, PR or SD, according to RECIST, which was maintained for at least four weeks from the initial manifestation of that rating. Patient visits were scheduled every three weeks and at the end of treatment to monitor safety, compliance and determine side effects. The safety assessment included documentation of the adverse events, clinical laboratory tests (hematological and biochemical analyses), physical examination and measurement of vital signs.

### Statistical analysis

This was an uncontrolled mono-institutional phase II trial. The primary endpoint of this trial was the progression-free survival (PFS) rate at four months. The secondary endpoints were: Overall tumor response (CR, PR and SD), TTP (initial treatment until PD) and overall survival (OS; initial treatment to mortality). The two-stages of Simon’s optimal design were used to test the null hypothesis (H0) that the PFS rate at four months was 20% against the alternative hypothesis (H1) of 60% With a sample size of 21 patients, this study had 80% power and an α level of 0.01. TTP and OS were estimated according to the Kaplan-Meier method. All the analyses were performed using Stata 11.0 (Stata Corporation, College Station, TX, USA).

## Results

### Patient characteristics

The baseline characteristics of the patients are shown in Table I. Initially, the ECOG performance status was 0 in 11 patients (50%) and 1 in 10 patients (50%). All patients had documented background chronic liver disease and 17 of the 21 patients had a CP classification of A. Considering viral infections, five patients were positive for HBV, 15 patients were positive for HCV and only one patient was positive for the two viruses, HBV/HCV. The α-fetoprotein range was 1–108 ng/ml (median, 41.6 ng/ml). Extrahepatic spread was present in only five patients (three bone and two lung), while portal vein thrombosis was observed in 11 patients (50%).

### Dose and duration of therapy

The median time of treatment was 4.5 months (range, 2–7 months). A total of 48.3 treatment cycles were administered (mean, 2.3 cycles for each patient; range, 1–3.6) and 11 patients (60%) received 100% of the planned study drug. Sorafenib was administered at a daily mean dose of 700 mg (range, 600–800 mg). For nine patients, the treatment was discontinued (45%) due to PD.

### Efficacy

All patients were considered evaluable for the primary endpoint. The PFS rate at four months was 70%. One patient (5%) achieved PR and 11 patients achieved SD (50%); however, no CR was reported. The DCR was 45% (Table II).

### TTP and OS

**Figure 1:**
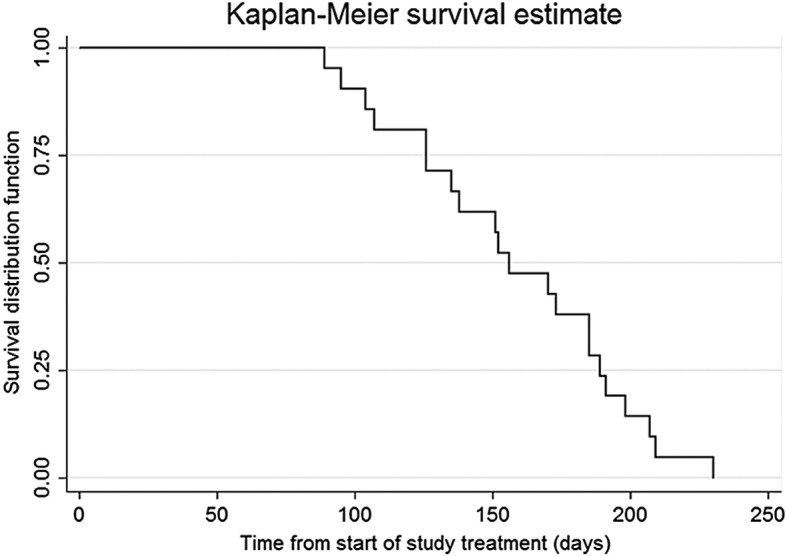
Kaplan-Meier estimate of the median time to progression was 5.2 months (95% confidence interval, 4.2–6.2 months).

**Figure 2:**
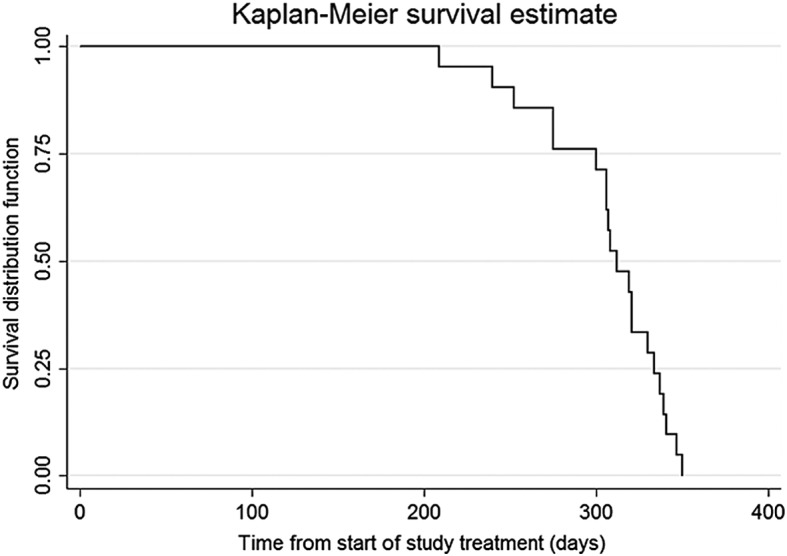
Kaplan-Meier estimate of the median overall survival was 10.4 months (95% confidence interval, 10–11 months).

The median TTP was 5.2 months [95% confidence interval (CI), 4.2–6.2 months] and the median OS time was 10.4 months (95% CI, 10–11 months) (Figs. 1 and 2).

### Toxicity

All patients were evaluable for toxicity. The overall incidence of treatment-related adverse events (any grade) was 80% (18 patients). The adverse events that were predominantly reported were grade 1 or 2; the most common adverse events were dermatologic, constitutional and gastrointestinal. Grade 1 and 2 toxicity included 20% hyperemia, 25% anorexia and 10% vomiting. Grade 3 toxicity included fatigue (5%), diarrhea (5%), HFSR (10%) and hypertension (5%). No grade 4 treatment-related toxicities were reported.

## Discussion

In this phase II trial, the combination of sorafenib and EHY was well tolerated and showed noteworthy antitumor activity; a four month PFS rate of 70% was reported, with 50% of patients achieving SD and 5% achieving PR. The DCR was 45% and the median TTP and OS time were 5.2 and 10.4 months, respectively. No grade 4 treatment-related toxicities were reported, while the most frequently reported grade 3 adverse events were similar to those reported in previous studies (fatigue, diarrhea and HFSR) (5,6).

These results compare favorably with the sorafenib phase II study conducted by Abou-Alfa *et al* (17) in patients with advanced HCC; three (2.2%) of the 137 treated patients achieved PR, eight (5.8%) achieved a minor response and 46 (33.6%) achieved SD for at least 16 weeks. The TTP and OS time were 4.2 and 9.2 months, respectively. In the SHARP phase III trial, sorafenib was found to improve the OS by 44% in patients with HCC (P=0.0006) versus the placebo group; the median OS was 10.7 months in sorafenib-treated patients compared with 7.9 months in those administered with the placebo. No significant difference was identified between the two groups in the median time to symptomatic progression (TTSP; 4.1 vs. 4.9 months, respectively; P=0.77). The median time to radiological progression was 5.5 months in the sorafenib group and 2.8 months in the placebo group (P<0.001). In total, 2% of patients achieved PR and 71% achieved SD. The DCR was 43%. The most commonly observed adverse events in patients receiving sorafenib were diarrhea, weight loss, HFSR and hypophosphatemia (5). In the phase III Asia-Pacific Liver Cancer Study, treatment with sorafenib was associated with a significantly longer OS time (median, 6.5 vs. 4.2 months for sorafenib and placebo, respectively; HR, 0.57; 95% CI, 0.42–0.79; P=.0005). The median TTSP was 3.5 months. A total of 3.3% of patients achieved PR and 54% achieved SD. The DCR was 35.3%. The most frequent grade 3/4 drug-related adverse events reported for sorafenib were HFSR (10.7%), diarrhea (6%) and fatigue (3.4%) (6). Since sorafenib was found to improve OS in these two phase III trials, sorafenib became the standard of care for advanced HCC; however, the benefits remain modest. Combining this drug with locoregional or systemic therapy may improve the outcome of advanced HCC patients.

As previously reported, experimental data for sorafenib indicate that hyperthermia inhibits angiogenesis and increases apoptosis. Furthermore, certain *in vitro* studies have shown that hyperthermia may alter the properties of metastatic potential in cancer cells and inhibit tumor metastasis due to the inhibition of hypoxia and TGF-β1-induced epithelial-mesenchymal transition in HepG2 HCC cells (17,19). The heat sensitivity of this tumor cell line decreases with rising percentages of the hepatic stellate LX-1 cell line (model of liver fibrosis) in coculture (20,21).

Few studies have analyzed EHY treatment in HCC and the efficacy of targeted therapy plus EHY remains unknown. Breast cancer *in vitro* and *in vivo* studies have indicated that mild hyperthermia sensitizes cancer cells to PARP-1 inhibitors (22,23).

In our previously reported study, the feasibility and safety of combining a chemical treatment (transarterial chemoembolization) with a physical treatment (radiofrequency ablation) in patients with hepatic malignancies was investigated. Therefore, we further hypothesized that combining a chemical systemic drug with a physical locoregional treatment may exhibit a synergistic effect (24). In the current study, a phase II trial was conducted in advanced HCC patients to evaluate whether EHY may potentiate the effect of sorafenib through reduction of angiogenesis and increased apoptosis.

These combinations act on the microenvironment of tumor cells. The multikinase inhibitory profile of sorafenib leads to effects in cancer cells, as well as the ECs and pericytes of tumor vasculature. First, EHY determines a energy absorption in the extracellular fluid and then, through a temperature gradient between the extracellular and the intracellular compartment, a destruction of tumor cells is observed (4,7).

The results of the present study showed that the sorafenib plus EHY combination is feasible and well tolerated; no major complications were observed. The initial findings suggested that this combination offers a promising option for advanced HCC, representing a new and challenging area for future clinical study. Further large studies are required to confirm these preliminary results.

## Figures and Tables

**Table I:** Patient baseline characteristics.

Characteristics	n (%)
Age, years	
Median	64
Range	55–73
Gender	
Male	14 (67)
Female	7 (33)
ECOG performance status	
0	11 (50)
1	10 (50)
2	0
Child-Pugh status	
A	17 (80)
B	4 (4)
Hepatitis virus status	
HBV infection	5 (20)
HCV infection	15 (75)
HBV/HCV infections	1 (5)
α-fetoprotein >ULN	
Yes	15 (75)
No	6 (25)
Macroscopic vascular invasion	
Yes	11 (50)
No	10 (50)
Extrahepatic spread	
Yes (bone and lung)	5 (25)
No	16 (75)

ECOG, Eastern Cooperative Oncology Group; HBV, hepatitis B virus; HCV, hepatitis C virus; ULN, upper limit of the normal range.

**Table II:** Response rates according to the Response Evaluation Criteria in Solid Tumors.

Response	n (%)
Complete response	0
Partial response	1 (5)
Stable disease	11 (50)
Progressive disease	9 (45)
Disease control rate	9 (45)
